# Curability of Hepatic Cirrhosis by Iodide of Potassium and Jaborandi

**Published:** 1890-10-11

**Authors:** 


### CURABILITY OF HEPATIC CIRRHOSIS BY IODIDE OF POTASSIUM AND JABORANDI.

Last year Dr. Lanceraux created a great impression in Paris by the publication of several cases of cirrhosis, or as it would be better called, chronic interstitial hepatitis, by iodide of potassium. We have, however, read of a similar case reported in, we believe, the *Arch. de Méd. Militaire* some years ago, by a French army surgeon in Algiers, and shortly before the appearance of M. Lancereaux's work Dr. O. Prados published in the *Rivista medica di Bojota* two interesting cases of severe chronic alcoholism, with cirrhosis, ascites, œdema of lower extremities, and pale jaundice, accompanied in one instance by vomiting and tremors, both of which were so far cured as to be relieved of all symptoms and to feel in excellent health after a month's treatment with iodide of potassium and jaborandi, and a strictly milk diet, when a light mixed diet was permitted, but all alcohol forbidden. One patient, relapsing into his former intemperate habits some nine months later on his return to Europe, was again attacked by his original symptoms, and treated differently, soon succumbed. This treatment is well worth further trial, but considering the uncertain action of pilocarpine given by the mouth, when it frequently fails to induce diaphoresis, even in dangerous doses, and the certainty and precision of its action when used subcutaneously, we would advise this mode of administration with the addition of digitalis to the iodide as a diuretic.

